# Adverse pathophysiological influence of early testosterone therapy on the testes of boys with higher grade sex chromosome aneuploidies (HGAs): a retrospective, cross-sectional study

**DOI:** 10.1007/s40618-020-01452-w

**Published:** 2020-10-24

**Authors:** M. Spaziani, C. Tarantino, C. Pozza, A. Anzuini, F. Panimolle, G. Papi, D. Gianfrilli, A. Lenzi, A. F. Radicioni

**Affiliations:** 1 Section of Medical Pathophysiology and Endocrinology, Department of Experimental Medicine, Sapienza University of Rome, 00161 Rome, Italy; 2 Centre for Rare Diseases, Policlinico Umberto I, Rome, Italy

**Keywords:** 48,XXXY, 48,XXYY, 49,XXXXY, Sertoli cells, Leydig cells, Cryptorchidism

## Abstract

**Purpose:**

Higher grade aneuploidies (HGAs) of the male sex chromosomes are a rare genetic group of pathologies caused by nondisjunction meiotic events. The aim of this study was to evaluate the impact of early androgenic therapy on the testicular secretory hormone profile, and the pathophysiological implications.

**Patients and methods:**

In this cross-sectional study, 18 HGA subjects aged 6–8 years were recruited. They were divided into two groups, based on whether or not they had previously undergone testosterone therapy (group 1: 11 untreated subjects; group 2: 7 treated subjects). Serum FSH, LH, testosterone (T), inhibin B (INHB) and anti-Müllerian hormone (AMH) were determined, and auxological parameters were assessed. Five group 1 patients and four group 2 patients were treated with hCG (human chorionic gonadotropin) for inguinal cryptorchidism; their hormone profile and auxological parameters were assessed both pre- and post-hCG treatment.

**Results:**

Group 1 subjects showed significantly higher testicular volume and higher levels of AMH and INHB (*p* < 0.0001). Subjects who had undergone hCG therapy showed a significantly higher testicular volume, penis length (respectively, *p* = 0.008 and *p* = 0.0005 for group 1 and *p* = 0.04 and *p* = 0.001 for group 2) and T (*p* = 0.005 for group 1 and *p* = 0.004 for group 2).

**Conclusions:**

HGA patients undergoing early testosterone therapy show an earlier and persistent suppression of testicular secretory function. At this age, the testes are still responsive to stimulation with hCG. The selection of patients to be treated must be accompanied by a thorough clinical and hormonal evaluation.

## Introduction

Higher grade aneuploidies (HGAs) of the male sex chromosomes were first described by Marco Fraccaro in 1960, in an article in The Lancet, when he described a 7-year-old boy with 49 chromosomes [1]. The most common HGA is the 48,XXYY form, with an incidence of 1:18,000–1:40,000 males [2]. It is caused by a paternal nondisjunction during the first or second meiosis, which leads to an XYY spermatozoon that fertilizes a normal oocyte. It is followed in frequency by 48,XXXY, which has an incidence of about 1:50,000 males [3]. This occurs when an XXY spermatozoon fertilizes a normal oocyte or when an XXX oocyte is fertilized by a normal spermatozoon. The HGA with the heaviest phenotypic impact is 49,XXXXY, with an incidence of around 1:85,000–1:100,000 males [3]. It is the result of a nondisjunction event during the first or second maternal meiosis, leading to the development of an aneuploid XXXX oocyte, fertilized by a normal spermatozoon [4]. The rarest HGA is 49,XXXYY, although its incidence is currently unknown, as only six cases have been reported in the literature [5]. It is the consequence of a normal oocyte fertilized by an XXYY spermatozoon, an XX oocyte fertilized by an XYY spermatozoon, or an XXX oocyte fertilized by a YY spermatozoon.

The most common significant clinical features of HGAs are dysmorphic facial features, hypotonic musculature, tremors, skeletal deformities, genital anomalies, hypergonadotropic hypogonadism and neurologic and cognitive impairment. Their severity tends to be worse in subjects with 49 chromosomes, who are generally also shorter [6–10].

A recent paper published by our group was the first to provide a detailed hormonal and metabolic evaluation of HGAs. We showed that these conditions are fully distinct from Klinefelter syndrome (KS) even though they share some features, such as testicular dysgenesis and hence hypergonadotropic hypogonadism. We demonstrated that HGA subjects show premature testicular damage, characterized by an earlier increase in gonadotropins and drop in testosterone (T) and inhibin B (INHB) [11].

There is considerable scientific literature on the hormonal physiology of testicular development, and especially on the relationship between Sertoli cell maturation and proliferation, anti-Müllerian hormone (AMH), INHB and T and their reciprocal and interdependent changes in concentration during childhood until the onset of puberty. Briefly, after birth, AMH is secreted by immature Sertoli cells independently of FSH levels, but FSH further increases AMH secretion by inducing Sertoli cell proliferation and upregulating AMH transcription [12, 13]. The sudden increase in circulating and intratesticular T in puberty induces the transcriptional suppression of AMH. This leads to a rapid decline in its concentration through interaction with the Sertoli cell androgen receptor (AR): the fact that the latter is not expressed until the first year of post-natal life explains why both AMH and FSH are elevated during mini-puberty [14–18]. Sertoli cells in the prepubertal testes produce both the α and β subunits of inhibin, so INHB, like AMH, is a marker of Sertoli cell activity in this phase [19].

Some articles have also been published in the last decade about the possible positive neurodevelopmental and body composition effects of androgen therapy administered during childhood and adolescence, especially on KS and, to a lesser extent, HGA patients [20–23]. However, there are very few or no references to the possible impact of such an early treatment on testicular maturation and development.

In view of the above, the aim of this study was to characterize the hormonal profile of the pituitary–gonadal axis in HGA boys and to determine whether, and to what extent, early testosterone therapy may modify the physiological interaction of the hormones responsible for testicular maturation.

## Patients and methods

### Subjects

This was a retrospective, cross-sectional study involving 18 pediatric HGA patients attending the Rare Disease Center at the Experimental Medicine Department of Sapienza University of Rome, Umberto I Policlinico. All patients had a post-natal genetic diagnosis, performed from a few months to a maximum of 2 years after birth. They had the following karyotypes, performed on at least 40 metaphases: three with 48,XXXY; eight with 48,XXYY; five with 49,XXXXY; one with 49,XXXXY (96)–48,XXXY (4) mosaicism and one with 49,XXXXY (12)–48,XXXY (88) mosaicism.

The patients were divided into two groups, based on whether they had previously undergone testosterone treatment or not. Eleven patients had never undergone testosterone therapy (group 1—untreated), while seven had received androgenic replacement therapy (group 2—treated) administered by specialists at a different hospital. The treatment regimens are reported in Table 1. These patients underwent clinical and hormonal assessments on coming to our attention, to compare them with group 1 subjects. These assessments were carried out at least 3 months after the last testosterone injection.

**Table 1:** Treatment time and type for group 2 subjects

Patient	Karyotype	Treatment type	Dosage	Age started	Duration
1	48,XXXY	Testosterone enanthate i.m	50 mg per month	4.7 years	3 months
2	49,XXXXY (96)–48,XXXY (4)	Testosterone enanthate i.m	25 mg per month	5 years	4 months
3	49,XXXXY	Testosterone enanthate i.m	25 mg per month	4.8 years	3 months
4	49,XXXXY	Testosterone enanthate i.m	25 mg per month	5.4 years	3 months
5	48,XXXY(88)–49,XXXXY(12)	Testosterone enanthate i.m	25 mg per month	5.7 years	3 months
6	49,XXXXY	Testosterone enanthate i.m	50 mg per month	3.3 years	3 months
7	48,XXYY	Testosterone enanthate i.m	25 mg per month	5.2 years	4 months

*i.m.* intramuscular

The statistical comparison between the two groups was done for the age range 6–8 years (≥ 6 and < 8), for two reasons. First, samples for the group 2 (treated) patients were available from when they were 6 years old, and second, this range avoided the inclusion of older subjects who already displayed clinical and hormonal signs of the onset of puberty. This decision led to the exclusion a priori of several HGA subjects outside the study age range, namely five untreated patients (three aged between 0.52 and 5.9 years, and two aged 8.3 and 12.2 years, respectively) and two treated patients (aged 9.3 and 11.8 years, respectively).

The mean age ± SD of the study population was 7.0 ± 0.8 years for group 1 and 7.2 ± 0.4 years for group 2. Five group 1 patients (45%) (mean age ± SD = 6.8 ± 0.6) and four group 2 patients (57%) (mean age ± SD = 7 ± 0.2) presented undescended testes (inguinal cryptorchidism). These patients were treated with hCG (chorionic gonadotropin) 2000 IU per week for 5 weeks. As cryptorchidism is very common with HGAs, we decided not to exclude these subjects from the study. However, the hormone concentrations measured immediately after the end of hCG therapy were obviously excluded from the statistical analysis. Instead, we carried out an intra-group comparison of hormonal profile and auxological data (testicular volume and penis length) pre- and post-hCG therapy (7 days after the last injection), and an inter-group, post-hCG, comparison.

### Hormone analysis

Baseline blood samples were obtained from all subjects by antecubital venous puncture in the early morning (7.30–9.00 a.m.) after an overnight fast for determination of serum concentrations of FSH, LH, T, INHB and AMH. Samples were centrifuged after 30 min and the serum immediately frozen at – 20 °C. All tests were performed in duplicate in the laboratory of the Department of Experimental Medicine (Section of Medical Pathophysiology), Sapienza University of Rome. Serum FSH, LH and T were measured by chemiluminescent microparticle immunoassay (CMIA, Architect System) (Abbott Laboratories, IL, USA), with limits of detection of 0.05 IU/L, 0.07 IU/L and 0.28 nmol/L, respectively. The intra- and inter-assay coefficients of variation were 3.1 and 4.7% at 3.2 IU/L (FSH), 3.6 and 5.1% at 3.3 IU/L (LH) and 2.1 and 3.6% at 10.08 nmol/L (T). The normal ranges for prepubertal subjects (i.e., all subjects included in the study) were < 0.05–2.00 IU/L (FSH), < 0.07–1.80 IU/L (LH) and < 0.28–2.2 nmol/L (T) [24–26]. Serum INHB was measured using an enzymatically amplified two-site two-step sandwich-type immunoassay (ELISA) (Beckman Coulter, Inc. Brea CA, USA). The limit of detection was 7.0 pg/mL and the intra- and inter-assay coefficients of variation were 3.3% and 7.2% at 122 pg/mL. The normal range for prepubertal subjects was 54.5–250.0 pg/mL [27]. Serum AMH concentration was measured using a Gen II enzyme-linked immunosorbent assay (ELISA) (Beckman Coulter, Inc. Brea CA, USA) with a limit of detection of 0.57 pmol/L; the intra- and inter-assay coefficients of variation were 5.6% and 7.5% at 94 pmol/L. The normal range for prepubertal subjects was 74–168 pmol/L.

To facilitate data processing, hormone concentrations below the limits of detection were set as follows: 0.03 IU/L for FSH, 0.04 IU/L for LH, 0.20 nmol/L for T and 4 pg/mL for INHB.

### Clinical and ultrasound evaluation

Anthropometric evaluations were performed using a stadiometer to measure height and standard scales to determine weight. Testicular volume was assessed by testicular colour Doppler ultrasound using linear transducers (5–12 MHz), through Lambert’s formula for the pediatric population, and the sum of the two volumes was considered for the statistical analysis. Finally, penis length was measured from the pubic ramus to the tip of the glans using a standard ruler.

### Ethics

All subjects were followed under a Regional Centre of Rare Diseases protocol approved as an integrated care pathway by both Policlinico Umberto I and the Lazio Regional Authority. Written informed consent was obtained from the participants’ parents.

### Statistical analysis

The statistical analysis was performed using Prism for Windows version 8 (GraphPad software, Inc.). Data in box plots are reported as median, minimum and maximum values (whisker). Unpaired two-tailed *T* tests or, when appropriate, non-parametric tests (Mann Whitney test) were used for comparisons after testing for normal distribution. Statistical significance was set at 95% confidence interval (*p* < 0.05).

## Results

All results are reported as mean ± SD.

### Comparison between the two groups

#### Auxological data

We did not find any significant differences in age, height, weight and penis length between group 1 and group 2. There was a statistically significant difference in testicular volume, which was higher in group 1 (Table 2).

**Table 2:** Clinical characteristics and hormone values of group 1 and 2 patients

	Group 1 (untreated)	Group 2 (treated)	*p* value
*N*	11	7	
Age (years)	7 ± 0.8	7.2 ± 0.4	NS
Height (cm)	127 ± 12	121 ± 6.6	NS
Weight (kg)	30 ± 7.5	26 ± 7.9	NS
Testicular volume (mL)	3.2 ± 1.3	1.4 ± 0.34	*0.01*
Penis length (cm)	4.5 ± 1.1	4.5 ± 0.71	NS
FSH (mIU/mL)	0.55 ± 0.27	0.49 ± 0.26	NS
LH (mIU/mL)	0.07 ± 0.05	0.04 ± 0.03	NS
T (nmol/L)	0.27 ± 0.1	0.52 ± 0.39	NS
INHB (pg/mL)	71 ± 22	17 ± 13	< *0.0001*
AMH (pmol/L)	149 ± 78	23 ± 22	< *0.0001*

Data are reported as mean ± SD

Italic values indicate *p* < 0.05

### Hormonal data

**Fig. 1:**
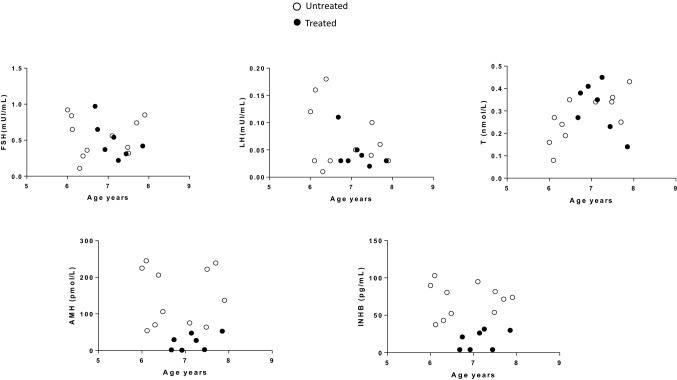
Gonadotropins, T, AMH and INHB levels against chronological age in group 1 (untreated—white circles) and group 2 (treated—black circles) patients

INHB and AMH were significantly higher in group 1 patients (Table 2, Fig. 1). In contrast, we did not find any significant differences in FSH, LH or testosterone levels (Table 2, Fig. 1).

### Effects of hCG therapy

#### Auxological data, intra-group comparison

Following hCG therapy, there was a statistically significant increase in testicular volume (group 1: 39%; group 2: 31%) and penis length (group 1: 23%; group 2: 26%) in both groups. The means, SDs and *p* values are reported in Table 3.

**Table 3:** Effect of hCG therapy on testicular volume, penile length and hormonal data of group 1 and 2 subjects

	Pre-hCG	Post-hCG		*p* value
*Group 1 (n = 5)*				
Age (years)	6.8 ± 0.6			
FSH (mIU/mL)	0.51 ± 0.23	0.42 ± 0.18		NS
LH (mIU/mL)	0.09 ± 0.03	0.05 ± 0.01		NS
T (nmol/L)	0.23 ± 0.1	1.4 ± 0.39		*0.005*
INHB (pg/mL)	63 ± 25	57 ± 21		NS
AMH (pmol/L)	109 ± 68	103 ± 52		NS
Testicular volume (mL)	2.6 ± 1.2	4.3 ± 0.89		*0.008*
Penis length (cm)	4.4 ± 0.83	5.4 ± 0.78		*0.0005*
*Group 2 (n = 4)*				
Age (years)	7 ± 0.2			
FSH (mIU/mL)	0.41 ± 0.15	0.24 ± 0.12	NS	
LH (mIU/mL)	0.08 ± 0.04	0.05 ± 0.03	NS	
T (nmol/L)	0.36 ± 0.05	1.7 ± 0.54	*0.004*	
INHB (pg/mL)	12 ± 8	10 ± 6	NS	
AMH (pmol/L)	19 ± 12	15 ± 9	NS	
Testicular volume (mL)	1.3 ± 0.37	1.7 ± 0.26	*0.04*	
Penis length (cm)	3.8 ± 0.37	4.8 ± 0.46	*0.001*	

Data are reported as mean ± SD

Italic values indicate *p* < 0.05

### Auxological data, inter-group comparison

After hCG therapy, group 1 subjects displayed both testicular volume (4.3 ± 0.89 vs 1.7 ± 0.26 mL; *p* < 0.001) and penis length (5.4 ± 0.78 vs 4.8 ± 0.46 cm; *p* = 0.04) significantly higher than group 2 subjects.

### Hormonal data, intra-group comparison

hCG therapy produced a significant increase in T in both groups (Table 3). We did not find any differences in the other hormones evaluated in the study (Table 3).

### Hormonal data, inter-group comparison

Hormonal comparison between groups 1 and 2, following hCG therapy, showed significantly increased levels of INHB and AMH, and reduced levels of FSH in the subjects belonging to group 1: 32 ± 21 vs 8 ± 1.3 pg/mL, *p* = 0.004 for INHB; 45 ± 28 vs 10.7 ± 2.4 pmol/L, *p* = 0.04 for AMH; 0.19 ± 0.11 vs 6.6 ± 4.2 mIU/mL, *p* = 0.01 for FSH.

## Discussion

To our knowledge, this retrospective, cross-sectional study is the first to show how early testosterone therapy affects testicular development in HGA patients. We do not aim to affirm whether or not such therapy is appropriate in these patients, but we merely want to identify any clinical signs and/or hormonal abnormalities that may be an indicator of impaired testicular maturation.

Current knowledge of the endocrinological and metabolic aspects of HGAs comes from our previous paper, which demonstrated early testicular damage compared to classic 47,XXY patients. This manifested through an earlier increase in gonadotropins and decrease in T and INHB, already evident in patients under 12 years [11]. The population of the present study further restricts the age range to boys aged 6–8 years. The choice of this age range was in part driven by the need for a statistically homogeneous comparison between groups 1 and 2, as explained above. However, it was also motivated by the findings of Young et al., who affirmed in their 2005 paper that by the age of 8 years more than 90% of Sertoli cells express ARs, which explains the early pubertal decline in AMH induced by rising intratesticular T concentrations despite the increase in FSH concentrations [12]. It is, therefore, conceivable that AR expression is lower in boys aged 6–8 years, albeit still sufficient to determine a reduction in AMH secretion in the presence of an increased testosterone concentration.

Our study found significantly lower concentrations of AMH and INHB in patients who had previously (aged between 3.3 and 5.4 years: see Table 1) received testosterone therapy, while testicular volume was significantly higher in untreated subjects. These results provide important insights.

It is widely debated whether Sertoli cells undergo direct FSH stimulation during childhood. Some studies suggested that the secretory activity of Sertoli cells in childhood might be disconnected from direct FSH stimulation, although it has been postulated that FSH acts indirectly by stimulating the proliferation of Sertoli cells and the upregulation of AMH transcription [12, 13, 28]. In contrast, in 2005, our group found a direct correlation between INHB and FSH concentrations during pubertal stage G1 and, more generally, during the initial stages of testicular maturation. We also demonstrated a strong biphasic relationship between INHB and FSH during pubertal development, with inversion of this relationship in the mid-late stages of puberty [29]. As in the present study, the testicular volume of untreated patients was significantly higher than in treated patients, we can assume that exogenous testosterone therapy may reduce (directly and/or through the reduction of FSH) the activity and/or proliferation of Sertoli cells. This itself is an essential factor for tubular component maturation and hence increased testicular volume, and thus explains the reduction in both INHB and AMH levels.

As already reported in other studies, the possibility that AMH secretion could be regulated through a partly different mechanism to INHB can be excluded for HGA subjects as well as KS subjects [26]. However, it should also be considered that several studies have shown that both germ cells and Leydig cells can express INHB [30, 31]. In the prepubertal testes, inhibin α-subunit and βB-subunit are both co-localized in Sertoli cells and interstitial cells, while in the adult testicles they are expressed in different cells: inhibin βB-subunit is found in sperm germ cells (from pachytenes to round spermatids) and to a lesser extent in Leydig cells, while inhibin α-subunit is only found in Sertoli cells [32–36]. INHB is also thought to regulate Leydig cell differentiation in embryogenesis [37]. Therefore, on the assumption that both Sertoli cells and Leydig cells contribute to INHB secretion, we could explain its reduction in treated subjects in part as the suppressive effect that exogenous testosterone exerts on Leydig cells. The reduced secretion of intratesticular testosterone might also contribute to the reduced maturation of the tubular compartment, further explaining the lower testicular volume of treated subjects.

It might seem surprising that the low AMH and INHB concentrations in treated subjects were not associated with a parallel increase in FSH. Moreover, we found no increase in FSH in untreated subjects either. This seems to conflict with our previous study, in which we found an earlier increase in FSH levels in HGA subjects under 12 than in KS subjects, reaching an average value of about 1.5 mIU/mL. This may be because the present study investigated a younger age group (6–8 years), whereas the rise in FSH tends to occur after the age of 8 years. In addition, the low FSH levels in treated subjects despite their low AMH and INHB values may be because pulsatile secretion of hypothalamic GnRH has not yet begun at this age, and hence their pituitary gonadotropic cells are still silent.

One of the treated patients (patient 5 in Table 1) showed much higher AMH and INHB levels than the others in this group, even those his age and treatment were the same. It is not easy to explain this particular hormonal response. A lower AR expression and activation in Sertoli cells might justify the higher AMH levels, while a reduced suppressive effect of testosterone on Leydig cells, might, as described above, explain the INHB concentrations. In any case, his mean AMH (41.46 pmol/L) and INHB (27.2 pg/mL) levels were still just 28% (AMH) and 38% (INHB) of those in untreated subjects (see Table 2). This boy also had a greater testicular volume and penis length than seen in the other treated subjects, with both parameters closer to those of the untreated group.

Having said all this, phenotypic variability is an essential aspect of HGAS as well as of classic 47,XXY KS. The explanation may, therefore, lie in the random genetic mechanisms underlying the inactivation of the X chromosome, in AR CAG triplet length variability, or in other unknown epigenetic mechanisms. In fact, even in everyday clinical practice with these rare patients, we have observed extremely variable psychological and clinical signs in the same karyotype. This phenotypic variability may well also involve the testes and their development.

In our opinion, the decision on whether or not to administer early testosterone therapy should be based precisely on this clinical variability. As supported by the results of the present study, patients should not be prescribed androgen therapy indiscriminately but should undergo a preliminary clinical and neurodevelopmental assessment, which should include a careful characterization of the hormonal profile.

Several studies in the scientific literature highlight the role of testosterone on brain development and maturation [38–41]. These studies show its importance in the cortical development of certain brain areas, such as the left lower parietal lobe, the temporal gyrus and the sulcus calcarinus, which are involved in the development of visuospatial, sensory-motor and behavioral abilities (especially during puberty). Furthermore, during mini-puberty testosterone seems to be essential for language development and neurobehavioral sexual differentiation. A unique study of the effect of androgen therapy on the neurodevelopmental profile of HGA subjects compared two groups of 49,XXXXY children [20]. The first consisted of 11 subjects aged between 8 months and 14 years, of whom ten received three injections of testosterone enanthate 25 mg in three consecutive months at a mean treatment age of 12 months and one was given 40 mg of testosterone enanthate. The second group consisted of ten children aged 27 months to 9 years, who received no therapy and served as a comparison group. Reassessment of three main domains, movement, verbal and auditory comprehension and vocabulary, at about 6 years of age revealed a statistically significant overall improvement in the treatment group. However, the authors reported various limitations with their study, including lack of baseline androgen measurement, having based the decision to treat purely on small penis size, and variations in the timing of androgen therapy, from newborns to 30-month-old boys.

As already stated, it is not our intention to establish whether or not early testosterone therapy may help these subjects and their many clinical and behavioral problems, but we do believe that careful patient selection is essential. Our data show that the natural evolutionary path of the gonad is undoubtedly affected by androgenic therapy in early childhood, while patient 5 shows that it is also crucial to take phenotypic variability into account. The possible effects on the maturing reproductive system should also be highlighted in the information sheet provided to the parents of these patients.

In this study, we also evaluated the effect of hCG therapy on HGA patients with cryptorchidism. Five untreated patients (45%) and four treated patients (57%) presented undescended testes in the inguinal canal. We found a significant increase in both testicular volume and penis length in both groups after 5 weeks of hCG therapy. There was also a parallel significant increase in T concentrations. In addition, similar to what previously observed in the first comparison between groups 1 and 2, we demonstrated that the subjects belonging to group 1 showed a greater increase, both of the testicular volume and of the penis length, following the treatment with hCG, compared to group 2 patients. This clinical data were validated by the hormonal data, since group 1 patients presented higher levels of INHB, AMH and lower of FSH. It should certainly be underlined that the outcome of these auxological and hormonal comparisons also depends on the baseline values, pre-hCG, that were certainly higher in the subjects belonging to group 1. Ultrasound examination showed at least a partial descent of the testicles in three untreated and in two treated patients, with a final position in the external inguinal ring or in the high scrotum. Two patients in each group presented a recurrence after hCG therapy was stopped, with partial or complete ascendance. This was probably due to an anatomical alteration linked to the syndrome itself, such as a short spermatic cord. In any case, these results are broadly in line with the other literature reports on hCG therapy and cryptorchidism [42].

Given our approach, we feel that the most important aspect emerging from the hCG treatment is that the testicles of HGA subjects are still responsive to gonadotropin stimulation at this age, since Leydig cells respond with a significant increase in T. This finding could even open the doors to another possibility, namely the replacement of testosterone therapy in clinical practice with a more “physiological" therapy involving gonadotropins to treat young HGA patients. Further studies are required to clarify this last aspect, especially in evaluating the efficacy of the two treatments in relation to psycho-cognitive improvement, while taking their different economic impacts into account.

## Conclusions

In conclusion, HGA patients undergoing early testosterone therapy show a reduced testicular volume and decreased testicular secretory activity and consequently face an earlier functional testicular suppression. We consider it essential to conduct a careful preliminary assessment, including a thorough hormone study, before opting for this treatment. Finally, it should be considered that the testicles of these children are still responsive to gonadotropin stimulation at this age, which could open up new, more physiological therapeutic options.

The future challenge will be to widen the clinical records of HGA subjects, to discriminate the “weight” of each different chromosomal arrangements on the clinical and hormonal manifestations.
